# Effects of Different Solvents on the Total Phenol Content, Total Flavonoid Content, Antioxidant, and Antifungal Activities of *Micromeria graeca L*. from Middle Atlas of Morocco

**DOI:** 10.1155/2024/9027997

**Published:** 2024-02-26

**Authors:** Fatima El Kamari, Hajar El Omari, Karima El-Mouhdi, Amina Chlouchi, Anjoud Harmouzi, Ilham Lhilali, Jihane El Amrani, Chadia Zahouani, Zouhair Hajji, Driss Ousaaid

**Affiliations:** ^1^Laboratory of Natural Substances, Pharmacology, Environment, Modeling, Health and Quality of Life, Faculty of Sciences Dhar EL Mahraz, Sidi Mohamed Ben Abdellah University, Fez, Morocco; ^2^Natural Resources Management and Development Team, Laboratory of Health and Environment, Faculty of Sciences, Moulay Ismail University, Meknes, Morocco; ^3^Ministry of Health and Social Protection, Higher Institute of Nursing Professions and Healthcare Techniques, Meknes, Morocco; ^4^Laboratory of Natural Resources and Sustainable Development, Ibn Tofail University, Kenitra, Morocco; ^5^National Higher School of Chemistry, IUT, Kenitra, Morocco; ^6^Agrophysiology, Biotechnology, Environment and Quality Laboratory, Sciences Faculty, Ibn Tofail University, Kenitra, Morocco; ^7^Cluster of Competence Environment and Health, Faculty of Sciences, Moulay Ismail University, Meknes, Morocco; ^8^Ministry of Health and Social Protection, Higher Institute of Nursing Professions and Healthcare Techniques, Fez, Morocco; ^9^Laboratory of Natural Resources and Economics of Sustainable Development, Polydisciplinary Faculty of Larach, Abdelmalek Essaadi University, Tetouan, Morocco; ^10^Economics and Management, Sidi Mohamed Ben Abdellah University, Fez, Morocco

## Abstract

*Micromeria graeca* L. is a dense chemical source of bioactive compounds such as phenolic compounds, which have various health-related properties. The current study aimed to investigate the impact of different extractor solvents on phenol and flavonoid contents, as well as the antioxidant and antifungal activities of different extracts. Initially, three extractor solvents (methanol, ethyl acetate, and water) were used to prepare the Soxhlet extracts, which were then examined for their polyphenolic content, flavonoid content, and antioxidant potential using three complementary assays (DPPH, FRAP, and TAC). The antifungal capacity against the two fungal strains (*Candida albicans* and *Aspergillus niger*) was performed using the method of diffusion on disc. The dosage of phytochemical compounds revealed that the highest values were established in water extract with values of 360 ± 22.1 mg GAE/g dry weight plant and 81.3 ± 21.2 mg RE/g dry weight plant for TPC and TFC, respectively. In addition, the strongest antioxidant activity measured by DPPH and FRAP assays was established in water extract with IC_50_ values of 0.33 ± 0.23 and 0.23 ± 0.12 mg/mL, respectively, while the methanol extract showed the best antioxidant activity as measured by TAC with an IC_50_ of 483 ± 17.6 mg GAEq/g dry weight plant. The water extract recorded the most important antifungal activity against *Candida albicans* with an inhibition zone of 16 ± 1.6 mm and MFC = 500 *μ*g/mL, whereas ethyl acetate extract showed the lowest activity against both studied fungi strains. *Micromeria graeca* L. contains considerable amounts of bioactive contents with high antioxidant and antifungal potentials, which may make it a promising source of antioxidants and natural antifungal agents.

## 1. Introduction

Medicinal plants occupy an important place in the daily diet and healthcare of several civilizations worldwide [1–3]. They are the main source of nutrients and nutraceuticals, which are implicated in the proper functioning of the body [1]. The beneficial properties of medicinal plants appeared in different writing as effective against several diseases, including diabetes, inflammation, cancer, wounds, and infectious diseases [4, 5]. Oxidative stress is the main factor leading to the development of various diseases. It is described as an imbalance in the production of reactive oxygen species (ROS) and the antioxidant defense system [6]. The antioxidant defense system corroborates the induction and progression of numerous diseases, such as neurodegenerative disorders [7], cardiovascular diseases [8], cancer [9], diabetes [10], and inflammatory diseases [11]. ROS are extremely reactive and they can attack cellular molecules, including proteins, lipids, and DNA, which leads to cellular damage. Phytochemicals contained in natural sources, such as medicinal plants would be a strong antioxidant to manage oxidative stress [12].

*Micromeria graeca* L. is an herbaceous plant belonging to the *Lamiaceae* family. It is a widespread species in the Mediterranean Basin, frequently used for therapeutic purposes and as a spice [13]. It is a wealthy vegetal matrix rich in bioactive compounds, which confirms its utility in traditional medicine to treat several diseases, such as diabetes, dyslipidemia, inflammation, cancer, and infectious diseases [14–17]. The administration of aqueous extract of aerial parts of the plant showed a significant decrease of arterial blood pressure in hypertensive animals [18]. In addition, the same extract found to be effective to reduce blood sugar level and total cholesterol level with significant increase of HDL-c level [14]. The beneficial properties of *M. graeca* are ascribed to its phytochemical content. Metabolomics profile of *M. graeca* essential oil revealed different components of bioactive compounds, including geranial (36.93%) z-citral (18.25%), 1,8-epoxy-p-menth-2-ene (13.01%), nerol (11.69%), and isoaromadendrene epoxide (10.14%) [15]. An attempt to explain the antifungal effect of geraniol revealed that this compound disturbs cell membrane integrity by interfering with ergosterol biosynthesis and inhibiting the PM-ATPase [19]. The extraction method is a pivotal step to extract the highest amounts of bioactive compounds. It has been found that drying methods and extractor solvents greatly affect the phytochemical profile of extracts [20, 21]. While the efficiency of the extraction process and suitability of solvents constitute a keystone to extract the highest amounts of bioactive components with health-related beneficial effects [22]. Several studies have revealed that several factors affect the yield of bioactive components recovery, such as the nature of the extractant, particle size, temperature, sample/solvent ratio, pH, and pressure, thereby affecting the biological properties of the extracts [23–25]. However, there is limited data on the antioxidant and antifungal properties of different *Micromeria graeca* extracts.

For this purpose, the main objective of the present study was to evaluate the antioxidant and antifungal activities of different extracts of *Micromeria graeca* L. originated from the Middle.

## 2. Materials and Methods

### 2.1. Plant Material

Aerial parts of *Micromeria graeca* were collected during flowering period (March 2020) in the Sefrou region (Middle Atlas of Morocco) (33°49′59″N 4°50′23″W) (Table 1). The plant was identified by the SNAMOPEQ laboratory team (Department of Biology, FSDM, USMBA, Fez, Morocco).

### 2.2. Soxhlet Extraction Technique

Before the extraction process, the aerial parts were washed and air-dried. Then the aerial parts were finely ground in a laboratory homogenized. The extraction method was performed using three different extractor solvents (water, methanol, and ethyl acetate) [22, 26], and the solid-to-liquid ratio was 1/20 using a Soxhlet extractor for 8 h. The rotary evaporator was used to concentrate all extracts and then stored in a refrigerator at 4°C.

### 2.3. Determination of Total Phenols Content

The total phenolic concentration (TPC) of the extracts was assessed by the Folin–Ciocalteu method [27]. Briefly, 0.5 mL of a known dilution of the extract and 2 mL of 7% sodium carbonate solution were added to 2.5 mL of 10% (v/v) Folin–Ciocalteu reagent. Optic density was measured at 760 nm (Jasco v-530) after 2 h of incubation in the dark. The finding was reported as milligrams of gallic acid equivalent per gram dry weight of the extract (mg GAE/g dry weight plant).

### 2.4. Determination of Total Flavonoid Content

The total flavonoid content (TFC) of the extracts was quantified using the aluminium chloride colorimetric assay [28]. Briefly, 1 mL of sample or standard solution was added to 10 mL volumetric flask containing 4 mL of distilled water. Then, 0.30 mL of 5% NaNO_2_ was added to the mixture and followed after five minutes by 0.3 mL of 10% AlCl_3_. Next, 2 mL of NaOH (1M) was added and the total was made up to 10 mL with distilled water. The solution was mixed and the optic density was measured against the blank at 510 nm (Jasco v-530). The finding was expressed as mg rutin equivalent per gram dry weight of each extract (mg RE/g dry weight plant).

### 2.5. Antioxidant Activities

#### 2.5.1. DPPH Radical Scavenging Assay

The radical scavenging activity of plant extracts against free radical DPPH was determined using the procedure reported by Blois [29], with slight modifications. The DPPH scavenging capacity was estimated using the following equation:

(1) $$\begin{array}{c} \text{Inhibition}\left(\%\right)=\left[\frac{\left(AC-AS\right)}{\text{AC}}\right]\times 100, \end{array}$$

where AC is the absorbance of the control and AS is the absorbance of the sample. The positive control used was BHT. The results were expressed as the IC_50_ (mg/mL). The IC_50_ values were calculated as the concentration causing a 50% inhibition of the DPPH free radical.

#### 2.5.2. Reducing Power Assay

The reducing power of *M. graeca* extracts was determined using the method of Oyaizu [30]. Various concentrations (1 mL) were added to phosphate buffer (2.5 mL, 0.2 M, pH 7.0) and 2.5 mL of 1% potassium ferricyanide (K3Fe (CN)). The mixture was then incubated at 50°C for 30 min. After incubation, 2.5 mL of trichloroacetic acid (10%) was added and the solution was centrifuged for 10 min. Finally, 2.5 mL of the supernatant was mixed with 2.5 mL distilled water and 0.5 mL FeCl3 (0.1%). The absorbance was measured at 700 nm (Jasco v-530). Quercetin was used as a standard. The results were expressed as IC_50_ (mg/mL). The IC_50_ (concentration corresponding to 0.5 of absorbance) was calculated by plotting the absorbance against the corresponding concentration.

#### 2.5.3. Total Antioxidant Capacity

The method was based on the reduction of Mo (VI) to Mo (V) and the consequent formation of a Mo (V) green phosphate complex at acidic pH [31]. A total volume of 25 *μ*L of extract was added to 1 mL of reagent solution (0.6 mol/L sulphuric acid, 28 mmol/L sodium phosphate, and 4 mmol/L ammonium molybdate). The mixtures were incubated at 95°C for 90 minutes, and then cooled to room temperature. Absorbance was measured at 695 nm (Jasco v-530). Total antioxidant activity was expressed as gallic acid equivalent number (mg GAEq/g dry weight plant).

### 2.6. Antifungal Activity

Antimicrobial activity of *Micromeria graeca* extracts was tested against *Aspergillus niger* (2CA932) and *Candida albicans* (ATCC 1026), isolated from patients in the intensive care rooms at the University Hospital Center, Fez, Morocco (CHU, Morocco). Fungal cultures were cultivated on PDA at 25°C for 7 days. The suspension of each fungus was made up in 0.85% normal saline. The turbidity of the fungal suspensions was then adjusted at 0.5% McFarland. An inoculated agar plate was prepared by transferring 20 mL of PDA to a sterile plate and uniformly covering the solid medium with 5 mL of soft agar, preinoculated with 100 *μ*L of fungal suspensions.

The antifungal efficacy of different extracts of *M. graeca* was investigated using the disc diffusion method [32]. Sterile filter discs (diameter 6 mm) were imbibed with 25 *μ*L of sample and placed on the medium. After incubation at 37°C for *C. albicans* and 30°C for *A. niger* for 24–48 h, the inhibition zone diameters were measured and reported as the mean ± SD of three repetitions for each fungal strain [15]. Amphotericin was used as a positive reference to determine the sensitivity of fungi species tested. The minimum inhibitory concentrations (MICs) of different extracts under study were realized in sterile 96-well microplate as described by Goullouce et al., with some modifications [33]. Briefly, the first well was filled with 200 *μ*L of PDA, while the other well were filled with 100 *μ*L of PDA and 25 *μ*L of each concentration of extracts. A series of dilutions of extracts is performed by successive dilutions of 1/2 in sterile distilled water, ranging from 1/2 to 1/512 of extract. 10 *μ*L of suspension of each microbe prepared in the same way previously described above was added to different wells. After the incubation of plates under the same conditions mentioned above, 40 *μ*L of triphenyltetrazolim chloride (0.5%) was added to different wells. Then, the plates were reincubated in the same conditions. MICs values were defined as the weakest concentration of the plant extract in which no growth was observed.

### 2.7. Statistical Analysis

Statistical analysis was carried out by one-way ANOVA followed by the Tuckey-test, using GraphPad Prism 5 Software. Differences at *p* <  0.05 were considered significant. The experiments were carried out in triplicate.

## 3. Results and Discussion

### 3.1. Effects of Solvent on Extraction Yield, Phenol Content, and Flavonoid Content

Table 2 summarizes the obtained results of yield, total phenolic content, and total flavonoid content. The treatment of results showed that yield values ranged between 3.2% and 47.2%. The highest yield value was recorded in water extract (47.2%), while the lowest yield value was registered in ethyl acetate extract (3.2%). It is worth noting that water was the most appropriate extractible solvent and showed a significant difference compared to other solvents (*p* < 0.05). Our results are in agreement with those evoked by Chew et al. [34]. Furthermore, subcritical water extraction is a potential engineering process that offers an environmentally acceptable solution for recovering diverse phytoactive compounds from different vegetal matrices [35]. In the same context, the extraction yield is highly affected by the extractor solvent's polarity [34]. The study conducted by Nawaz et al. found that extraction yield in highly polar extractants produced a higher recovery proportion with lower bioactive compound contents as compared to nonpolar extractants [36]. Researchers found that polar solvents with hydroxyl groups, such as water and methanol, are more effective in recovering solid mass from vegetal matrices [37]. The polarity indexes of the various extractor solvents utilized were 9, 6.6, and 4.3 for water, methanol, and ethyl acetate, respectively, resulting in a proportional extraction yield to the solvent polarity [38]. Indeed, extraction yield can also be influenced by several factors, including the chemical nature of the phytochemicals, the extraction method used, the particle size of the sample, and the existence of interfering compounds [39, 40]. While the combination of solvents with different polarities could increase the bioactive compound extraction efficiency with significant biological properties.

As shown in Table 2, the total phenolic content of the different extracts under study ranged between 211 ± 11.9 mg GAE/g and 360 ± 22.1 mg GAE/g. The highest TPC amount was recorded in water extract (360 ± 22.1 mg GAE/g), while the weakest quantity was found in ethyl acetate extract (211 ± 11.9 mg GAE/g). Statistical analysis showed a significant difference between all extracts under study (*p* < 0.05). Therefore, from the results displayed in Table 1, water was the suitable solvent for recovering of phenolic content. The study by Nawaz et al. found that the amount of total extractible compounds increases depending on the polarity of the solvent. The authors of the same study found a significant negative correlation between phenolic content and solvent polarity [36]. Furthermore, the extraction solvents used have been found to be implicated in 21.8% of total activity variation of different extracts [37]. Polar solvents were preferred to recover polar molecules, including polyphenols combined with carbohydrates [41]. The obtained results are in line with those reported by Ousaaid et al. registered the highest amount of TPC in aqueous extract (40.16 ± 0.25 mg GAE/g dry weight plant) [40]. In addition, the findings reported by Brahimi et al. are higher than those registered in the present study [42]. Vegetal matrix contains a wide range of bioactive components. The selection of the right and efficacious extraction method constitutes a determinant factor to predict the beneficial properties of the extract under study [43–45]. It has been found that the phytochemical profile of plants is highly influenced by several biological factors, such as genotype, and organ, ontogeny, as well as edaphic and climatic conditions (temperature, salinity, water stress, and light intensity) [46]. Furthermore, the type of solvent, the degree of polymerisation of the phenolic components, and their interaction all influence phenolic compound solvability [47, 48].

Concerning the total flavonoid content, the values of TFC changed in the range of 12.53 ± 1.17 to 81.3 ± 21.2 mg RE/g dry weight plant. The highest value was found in the water extract. Whereas, the lowest value was recorded in the ethyl acetate extract. Consequently, the analysis of the results obtained revealed that water was significantly the most appropriate solvent for extracting the bioactive ingredients from the plant under study. The obtained results from this study are higher than those reported by Fatiha et al. (2.4 ± 0.1 mg quercetin equivalent/g) [49].

### 3.2. Antioxidant Activity

Antioxidant activities of different extracts (water, methanol, and ethyl acetate) prepared from *M. graeca* were tested by three complementary assays, including DPPH· radical scavenging, iron (III) to iron (II) reducing activity, and the phosphomolybdenum assay.

The DPPH test, based on single-electron transfer mechanisms, is a simple and popular test for the assessment of antioxidant activity [50]. The IC_50_ values presented in Table 3 indicate that methanol extract exhibited an interesting antioxidant activity against free radical DPPH with an IC_50_ of 0.27 ± 0.25 mg/mL, followed by water extract with an IC_50_ value of 0.33 ± 0.23 mg/mL. The DPPH assay demonstrates the ability of the polar extract to donate hydrogen and/or electrons, which is an important factor for protecting biomolecules against negative effects of ROS. A positive correlation was observed between antioxidant activity and phenolic and flavonoid contents (Table 4). The obtained results are in line with those of Do et al. [41]. The authors found that methanol extract exhibited the highest antioxidant activity than aqueous extract [41]. It has been found that higher extraction yield may not result in high antioxidant extraction because of the presence of various molecular-weight hydrosoluble carbohydrate complexes, including glycosides that diminish the antioxidant abilities [37, 51].

Several studies have evaluated the antiradical activity of polar extracts of other Micromeria species. The results showed that the acetone extract of Egyptian *Micromeria nervosa* possessed a significant antioxidant activity compared the standard antioxidant (*α*-tocopherol) [52]. In addition, ethanol extracts of three species of *Micromeria*, including *M. croatica*, *M. juliana*, and *M. thymifolia*, naturally growing in Croatia and the methanol extract of *M. fruticosa* ssp., serpyllifolia from Turkey, showed significant DPPH- scavenging activity [53]. However, *M. croatica* was the least potent species with IC_50_ of 4.7 *μ*g/mL [53]. According to Vladimir-Knezevic et al., the anti-free radical activity of Micromeria species depends primarily on their chemical composition, including rosmarinic acid as a strong antioxidant agent [54].

Concerning ferric reducing power, aqueous extract exhibited the strongest activity than other extracts with an IC_50_ value of 0.23 ± 0.01 mg/mL, followed by methanol extract. However, all the extracts had a lower reducing power than the synthetic antioxidant quercetin (0.033 ± 0.006 mg/mL). The reducing capacity of *M. graeca* extracts may be due to its dense chemical composition, which is responsible for stabilising and blocking free radicals [53].

Total antioxidant capacity assay revealed that methanol extract was the most active with a value of 483 ± 17.6 mg AAE/g. Consequently, the differences between the different extracts are mainly due to the change in polarity of the solvent, which modifies their ability to dissolve a group of antioxidant substances [55].

### 3.3. Antifungal Activity

Table 5 displays the obtained results of the antifungal activity of *M. graeca* against two fungal strains. The treatment of the obtained results showed that both strains were sensitive to all extracts with range of diameter of inhibition between 8 mm and 16 mm. The aqueous extract was the most effective extract against both fungal strains compared to the methanol and ethyl acetate extracts (Table 3). In contrast, ethyl acetate had the lowest antifungal activity with a diameter of inhibition of 9 mm against *C. albicans* and 8 mm against *A. niger*. The obtained results from the present study are lower than those evoked by Benali et al. [15]. The authors found that *M. graeca* essential oil had a powerful antifungal effect against *C. albicans* with a zone inhibition of 44.33 ± 0.57 mm [15]. Furthermore, the aqueous extract of *M. graeca* exhibited an excellent inhibitory effect of aflatoxin production by *Aspergillus flavus* throughout molecular inhibitory mechanism [56]. The exploration of molecular mechanism revealed that bioactive components of the plant downregulated the *aflR* and *aflS* encoding for internal cluster ca-activators that control the aflatoxin biosynthesis [15]. The delve into the phytochemistry of *M. graeca* showed a broad spectrum of bioactive compounds, including geranial and z-citral [15, 57, 58]. The exploration of the antifungal activities of phytochemical compounds revealed their ability to act in a dose-dependent manner by exfoliation of the surface layer of fungi and disorganization of cell organelles [59]. Furthermore, the extract phenolic content, antioxidant and antifungal activities have a direct relationship, and the antioxidant and antifungal effects could be influenced by the phytochemistry composition of the extracts [59].

## 4. Conclusion

The present study was conducted to determine the effect of extractor solvent on extraction yield, phenolic and flavonoid content, antioxidant activity, and antifungal activity of *Micromeria graeca* from the Middle Atlas of Morocco. The results showed that the nature of the solvent used plays an important role in the yield of the extract and the total phenol and flavonoid contents. The highest phenolic and flavonoid contents were established in water extract, which expresses a high antiradical effect and the best antifungal activity against the fungi tested. *Micromeria graeca* can be suggested as a natural antioxidant and antimicrobial product for food preservation and the management of phytopathogenic fungi. Further in vivo studies are required to delve into the phytochemistry and molecular inhibitory mechanism of action of *M. graeca*.

## Figures and Tables

**Table 1:** Characteristics of sampling region.

Latitude	Longitude	Altitude (m)	Rainfall (mm/year)	Annual temperature (°C)	Bioclimatic stage
33°49′59″N	4°50′23″W	850	660	14.7	Semiarid

**Table 2:** Extraction yield, total phenolic content, and total flavonoid content of different extracts.

Extract	Yield (%)	TPC (mg) GAE (g)	TFC (mg) RE (g)
Water	47.2 ± 0.2^a^	360 ± 22.1^a^	81.3 ± 21.2^a^
Methanol	9.6 ± 0.3^b^	314 ± 19^b^	74.20 ± 14.51^b^
Ethyl acetate	3.2 ± 0.1^c^	211 ± 11.9^c^	12.53 ± 1.17^c^

Data are expressed as mean ± SEM of tree measurements. Different letters symbolized significant differences (*P* < 0.05) by mean of the nonparametric Turkey-test.

**Table 3:** Antioxidant activity of *Micromeria graeca* extracts.

Sample	IC_50_-DPPH (mg/ml)	Reducing power (mg/ml)	TAC (mg AAE/g)
Water	0.33 ± 0.23^a^	0.23 ± 0.12^b^	372 ± 22.5^b^
Methanol	0.27 ± 0.25^b^	0.28 ± 0.04^a^	483 ± 17.6^a^
Ethyl acetate	0.46 ± 0.11^c^	0.44 ± 0.01^c^	348 ± 32.7^b^
BHT	0.12 ± 0.01^d^	—	—
Quercetin	—	0.033 ± 0.006^d^	—
Gallic acid	—	—	219 ± 23.5^c^

Data are expressed as mean ± SEM of tree measurements. Different letters in each column symbolized significant differences (*P* < 0.05) by mean of the nonparametric Turkey-test.

**Table 4:** Correlation between *M. graeca* different extracts antioxidant activities and total phenolic (TPC) and total flavonoid contents (TFC).

Assays	TPC	TFC
DPPH	0.85^*∗∗*^	0.76^*∗*^
FRAP	0.54	0.37
TAC	0.49	0.41

^*∗*^Correlation is significant at the *P* < 0.05 level. ^*∗∗*^Correlation is significant at the *P* < 0.01 level.

**Table 5:** Antifungal activity of *Micromeria graeca* extracts against *Candida albicans* and *Aspergillus niger* with inhibition zones of growth (mm) and the minimum fungicidal concentrations (MFC).

Tested strains	Water		Methanol		Ethyl acetate		Amphotericin
	DI	MFC	DI	MFC	DI	MFC	DI
*C. albicans* (ATCC 1026)	16 ± 1.6	500	12 ± 0.33	4000	9 ± 0.66	4000	20
*A. niger* (2CA932)	12 ± 0.66	500	8 ± 0.66	4000	8 ± 0.33	4000	32

## Data Availability

The data used to support the findings of this study are included in the article.
